# The safety and effectiveness of music medicine as an intervention for depression: A systematic evaluation and re‐evaluation

**DOI:** 10.1002/brb3.3629

**Published:** 2024-09-11

**Authors:** Dayuan Zhong, Hui Cheng, Zhenghua Pan, Yumei Liu, Pingwen Liu, Jiarong Li, Jiaqi Chen, Yihui Deng, Xueming Ou, Huanjie Li, Xiangbo Kong

**Affiliations:** ^1^ Nanhai Hospital of Traditional Chinese Medicine Jinan University Foshan China; ^2^ Institute of Traditional Chinese Medicine Jinan University Guangzhou China; ^3^ Graduate School Guangzhou University of Chinese Medicine Guangzhou China; ^4^ Institute of Integrative Medicine Hunan University of Chinese Medicine Changsha China; ^5^ Department of geriatrics Foshan Hospital of Traditional Chinese Medicine Foshan China

**Keywords:** depression, music, music medicine, overview, overview of systematic reviews, systematic evaluation and re‐evaluation

## Abstract

**Background:**

As the methodological quality and evidence level of the existing systematic reviews (SRs) on music as an intervention for depression have not been thoroughly evaluated, a systematic evaluation and re‐evaluation (SERE) was conducted.

**Methods:**

Multiple databases including PubMed, Web of Science, Embase, China National Knowledge Infrastructure, SinoMed, Wanfang, and the VIP database were searched for SRs and meta‐analyses (MAs) on the effectiveness of music as an intervention for depression. The literature screening, evaluation of methodological quality, and assessment of evidence level were carried out by a team of researchers. The methodological quality was evaluated using the Assessment of Multiple Systematic Reviews 2 (AMSTAR 2) scale in accordance with the 2020 Preferred Reporting Items for Systematic Reviews and Meta‐Analyses (PRISMA) guidelines, and the Grading of Recommendations, Assessment, Development, and Evaluation (GRADE) criteria were utilized to assess the level of evidence.

**Results:**

A total of 18 SRs were included in the analysis. The 2020 PRISMA guidelines were utilized to evaluate various aspects such as search terms, funding sources, statistical methods for missing values, subgroup and sensitivity analyses, certainty assessment, excluded literature citations, assessment of publication bias, protocol information, conflicts of interest, and data availability, which were rarely reported. The evaluation of the studies using the AMSTAR 2 scale revealed that one article was rated as high quality, six were rated as low quality, and 11 were rated as very low quality. Based on the GRADE criteria evaluation, the quality of the evidence was found to be inconsistent, with reports primarily consisting of medium‐quality evidence.

**Conclusion:**

The methodological quality of SRs/MAs of music as an intervention in depression is generally poor, and the level of evidence is generally low.

## Graphical abstract

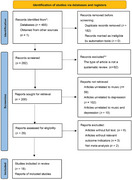

## 1 INTRODUCTION

Depression, also known as depressive disorder, is characterized by core symptoms such as low mood and loss of interest that are not proportional to the situation (Monroe & Harkness, 2022). Additional symptoms may include anxiety, agitation, hallucinations, and delusions (Monroe & Harkness, 2022). This mental health condition is highly prevalent and carries a high risk of mortality and disability (Benasi et al., 2021). Globally, 5% of adults experience depression annually, resulting in significant social and economic losses for individuals, families, communities, and countries (Herrman et al., 2022). Before the COVID‐19 pandemic, depression‐related economic losses were estimated to be approximately $1 trillion per year (Herrman et al., 2022). Although the etiology and pathogenesis of depression are not fully understood, research suggests that they may involve central nervous inflammation, intestinal microecological destruction, neurotransmitter abnormalities, and hypothalamic–pituitary–adrenal axis disorders (Hao et al., 2022; He et al., 2022).

At present, depression is treated through two main approaches: drug therapy and nondrug therapy. Western and traditional Chinese medicine are both effective in treating depression through drug therapy. However, antidepressants used in Western medicine may have problems such as poor efficacy, high recurrence rate, and strong adverse reactions (Fournier et al., 2010; Thase & Denko, 2008). The therapeutic effects and mechanisms of antidepressants used in Chinese medicine are unclear (Hao et al., 2022). Nondrug therapies such as physical therapy and psychotherapy are also commonly used. Physical therapy includes modified electroconvulsive therapy, repetitive transcranial magnetic stimulation, deep magnetic stimulation, magnetic convulsive therapy, transcranial direct current stimulation, low‐frequency magnetic stimulation, vagus nerve stimulation, deep brain stimulation, and phototherapy (Cui et al., 2021; Q. S. Tang, 2022). Among these, nonconvulsive electroconvulsive therapy is the most widely recognized nondrug treatment, which can be combined with antidepressant drug therapy for additional benefits. However, nonconvulsive electroconvulsive therapy also has problems such as uncertain clinical efficacy and adverse reactions (G. P. Zhong, 2020). Psychotherapy includes cognitive therapy, psychological support therapy, and mindfulness decompression therapy (Hu et al., 2022; Pampallona et al., 2004), with cognitive therapy being the most common type of psychotherapy (Hu et al., 2022). The use of cognitive therapy is limited due to its long treatment duration and lack of coverage by medical insurance companies in China, which leads to a high cost for patients (Ma et al., 2021).

Music has been widely used in research and clinical applications in Western countries due to its long history (Llovet, 2017). Music has the ability to induce positive emotions and relax the body and mind, and its antidepressant effect may be mediated by its impact on serotonin transmission and hippocampal brain‐derived neurotrophic factor levels in the central nervous system (Lin et al., 2011). Previous studies have confirmed the positive therapeutic effects of music on depression (Feneberg et al., 2021; Fu et al., 2023; Wall et al., 2023; X. Wang et al., 2023; Xue et al., 2023). Music‐based interventions can be broadly categorized into two types: music therapy and music medicine. Music therapy is a systematic intervention process facilitated by a certified music therapist who, in an evidence‐based manner, utilizes specially designed musical forms and therapeutic relationships formed during the process to assist the recipients in achieving mental and physical health goals (Chen & Gao, 2022). Music medicine involves interventions primarily focused on music listening provided by nonqualified individuals (Aalbers et al., 2017). Consequently, the standards for music medicine are broader than those for music therapy, making it more accessible in clinical treatments and everyday life. Moreover, music medicine is characterized by its simplicity, low cost, minimal resource requirements, and greater acceptance among individuals with depression (Werner et al., 2017).

Music medicine intervention for depression is not a novel concept. In fact, extensive clinical research has been conducted worldwide. The number of systematic reviews/meta‐analyses (SRs/MAs) based on the results of these clinical studies is also considerable. SRs/MAs, as a crucial research method, serve as the cornerstone for evaluating clinical effectiveness, formulating clinical guidelines, and standardization. It is also a significant source of evidence in evidence‐based medicine (Li & Li, 2008). Simultaneously, low‐quality SRs/MAs can mislead clinical decisions. Systematic evaluation and re‐evaluation (SERE), also known as Umbrella Review, stands at the top of the evidence‐based medicine pyramid (Huang et al., 2023; D. Y. Zhong et al., 2022). SERE, based on the results of SRs/MAs, rigorously integrates and evaluates the evidence information reported in previous SRs/MAs with caution. Its evaluation results can provide valuable references for clinical decision‐making and offer strong guidance. SERE is equally applicable to the field of music medicine intervention in depression. The quality, methodology, and evidence of previously published SRs/MAs reports on music medicine intervention for depression have not been systematically re‐evaluated. This study aims to re‐evaluate SRs/MAs on music medicine intervention in depression, providing more comprehensive evidence in evidence‐based medicine for clinical decisions on music medicine intervention in depression.

## 2 MATERIALS AND METHODS

### 2.1 Research registration

The research protocol for the umbrella review was written and registered through the PROSPERO website. The registration number is CRD42022376072.

### 2.2 Inclusion criteria

- The subjects of the study are patients with depression. The type of depression is not restricted, and both primary and secondary depression can be included. The treatment group focuses on music‐based interventions, which may be combined with or without antidepressant medication. Music therapy is not included in this.
- The control group consists of conventional treatment groups, which may be combined with or without antidepressant medication. However, the study should disclose that there is no significant difference between the combined treatment in the experimental group and the treatment in the control group.
- In addition, assuming the included articles are network MAs, they may contain various forms of music interventions, but we only extract the part related to music medicine. Other forms of music interventions are not included.
- The observational indicators mainly include depressive symptoms, effective rate, Hamilton depression scale, self‐rating depression scale, and other related indicators for patients with depression.
- The included studies are limited to SRs/MAs, excluding other types of reviews and articles.
- The language of the study is limited to Chinese and English, excluding other languages.
- The publication date of the SRs/MAs should be on or before November 12, 2022.

### 2.3 Exclusion criteria

- Exclude articles that do not meet the requirements of the study type. For example, articles that are not SRs/MAs. Detailed examples include clinical studies, basic research, case reports, reviews, and guidelines.
- Exclude articles with interventions that do not meet the requirements. For example, studies where the control group does not involve music‐based interventions or where music therapy is utilized. There are few restrictions on the interventions of the control group using conventional therapy, but it is required that the conventional therapy added to the control group and the observation group be basically consistent and comparable. If the conventional therapies in the two groups are not comparable, they will also be excluded.
- The population observed in this study consists of patients with depression, whether primary or secondary depression is included. Articles that do not include patients with depression or exclude depression will be excluded.
- In this study, we have predesigned some observational indicators. Articles that do not include these indicators will also be excluded.
- In addition to the four requirements mentioned above, articles with completely duplicated content or articles that cannot be accessed in full will also be excluded.

### 2.4 Retrieval methods and strategies

Databases used were PubMed, Web of Science, Embase, China National Knowledge Infrastructure (CNKI), SinoMed, Wanfang, and VIP (VIP China Science and Technology Journal Database, https://www.cqvip.com/). Search terms used were music, depression, SR, and MA. All studies published from the establishment of the database to November 12, 2022, were included. The search strategy included terms that were free words and subject words. The search process for the Web of Science database was as follows:

#1 music
#2 depression
#3 meta‐analysis
#4 systematic review
#5 #3 OR #4
#6 #1 AND #2 AND #5

### 2.5 Data extraction and quality evaluation

Two authors retrieved and filtered the articles, and then extracted and reviewed the data. Disagreements were resolved through negotiation with a third author. If any data were missing, corresponding authors were contacted for additional information. Articles that remained incomplete were excluded. The methodological and level‐of‐evidence quality of the included papers were assessed by four authors using the 2020 Preferred Reporting Items for Systematic Reviews and Meta‐Analyses (PRISMA) guidelines, the Assessment of Multiple Systematic Reviews 2 (AMSTAR 2), and the Grading of Recommendations, Assessment, Development, and Evaluation (GRADE) (Guyatt et al., 2008; Page et al., 2021; Shea et al., 2017). Following the evaluation, another author checked the data.

## 3 RESULTS

### 3.1 Search results and basic characteristics of the included studies

**FIGURE 1:**
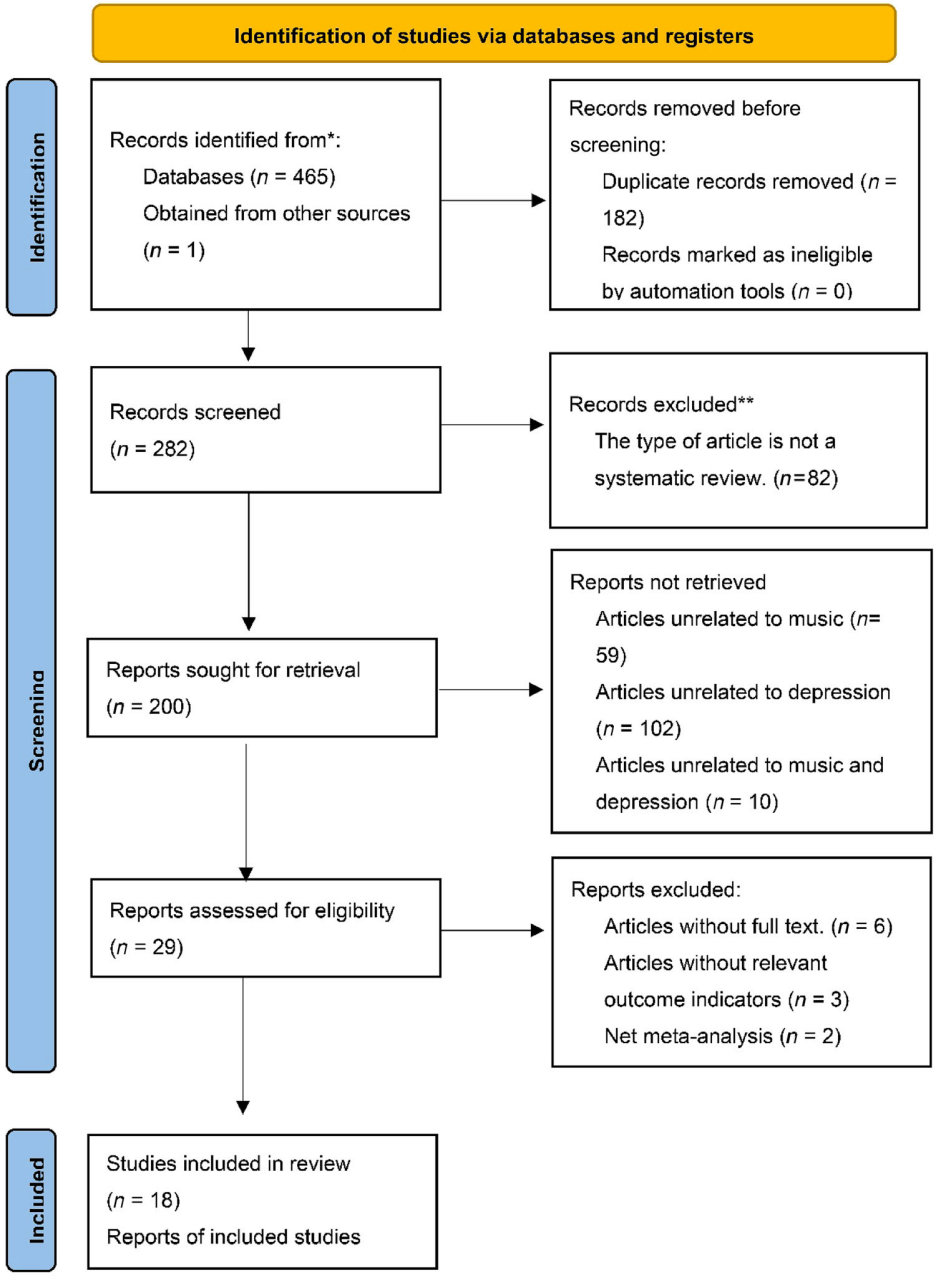
Flow chart of literature screening.

After a search of seven databases, 466 articles were obtained for review. Overall, 35 articles were retrieved from CNKI, 19 from Wanfang, 9 from VIP, 22 from SinoMed, 88 from PubMed, 129 from Web of Science, 163 from Embase, and 1 from another source. Using the E‐study software of CNKI, 182 duplicate articles were excluded. The authors excluded an additional 82 non‐SRs, 59 music‐unrelated articles, 102 depression‐unrelated articles, and 10 music‐unrelated and depression‐unrelated articles by reading the titles. After the full text of the literature was completed, six articles that could not be obtained in the full text, three articles without relevant outcome indicators, and two articles that were not SRs were excluded. Ultimately, 18 SRs, which represented a time span from 2014 to 2022, were included (X. F. Dai et al., 2015; Y. Q. Dai et al., 2016; Dayuan et al., 2022; Fan et al., 2015; Ji et al., 2015; Liu & Ji, 2021; Q. Tang et al., 2020; Tsai et al., 2014; Wan et al., 2018; X. N. Wang & Lin, 2017; Y. X. Wang et al., 2020; Yan et al., 2019; Yang et al., 2019; Yu et al., 2020; Zhang et al., 2022; Zhao et al., 2016; Zhu et al., 2021; Zou et al., 2017). The specific retrieval and screening process are shown in Figure 1. Four of the included studies used the Jadad score to evaluate the quality of the included SRs (X. F. Dai et al., 2015; Fan et al., 2015; Tsai et al., 2014; Wan et al., 2018); one SR did not specify a tool for evaluation of literature quality (X. N. Wang & Lin, 2017); and the remaining SRs used the Cochrane risk‐of‐bias tool for evaluation of literature quality (Y. Q. Dai et al., 2016; Dayuan et al., 2022; Ji et al., 2015; Liu & Ji, 2021; Q. Tang et al., 2020; Y. X. Wang et al., 2020; Yan et al., 2019; Yang et al., 2019; Yu et al., 2020; Zhang et al., 2022; Zhao et al., 2016; Zhu et al., 2021; Zou et al., 2017). Two SRs included non‐randomized, controlled trials for systematic evaluation (X. F. Dai et al., 2015; Tsai et al., 2014), as shown in Table 1 (X. F. Dai et al., 2015; Y. Q. Dai et al., 2016; Dayuan et al., 2022; Fan et al., 2015; Ji et al., 2015; Liu & Ji, 2021; Tsai et al., 2014; Wan et al., 2018; X. N. Wang & Lin, 2017; Y. X. Wang et al., 2020; Yan et al., 2019; Yang et al., 2019; Yu et al., 2020; Zhang et al., 2022; Zhao et al., 2016; Zhu et al., 2021; Zou et al., 2017).

**TABLE 1:** Basic characteristics of the included studies.

Study	Types of included studies	Quality evaluation	Number of included studies	Sample size	Therapy group	Control group	Conclusion
Tsai et al., 2014	RCT/N‐RCT	Jadad	8	NA	Music medicine plus standard treatment	Standard treatment	Music medicine moderately and significantly reduced depression in cancer patients
Fan et al., 2015	RCT	Jadad	19	1680	Music medicine plus Conventional treatment	Conventional treatment	Music medicine may alleviate depressive symptoms in cancer patients
X. F. Dai et al., 2015	RCT/CCT	Jadad	9	784	Music medicine plus Conventional treatment	Conventional treatment	Five‐element music has a certain auxiliary effect on reducing depressive symptoms in patients with post‐stroke depression.
Ji et al., 2015	RCT	CRT	9	581	Music medicine	Quiet environment	Music medicine can relieve college students ' depression
Y. Q. Dai et al., 2016	RCT	CRT	4	322	Music medicine plus Conventional treatment	Conventional treatment	Five‐element music for depression in patients with cardiovascular disease
Zhao et al., 2016	RCT	CRT	19	2,692	Music medicine plus standard treatment	Standard treatment	Music medicine has an effect on reducing depressive symptoms to some extent.
X. N. Wang & Lin, 2017	RCT	NA	8	852	Music medicine plus routine nursing	Routine nursing	Music medicine can significantly improve the depression of breast cancer patients
Zou et al., 2017	RCT	CRT	10	892	Music medicine plus Conventional treatment	Conventional treatment	Five‐element music has a certain auxiliary effect on reducing depressive symptoms in patients with post‐stroke depression.
Wan et al., 2018	RCT	Jadad	16	1431	Music medicine plus Conventional treatment	Conventional treatment	Music medicine combined with conventional therapy is superior to conventional therapy in reducing HAMD score of PSD patients.
Yan et al., 2019	RCT	CRT	12	1013	Music medicine plus routine nursing	Routine nursing	Music medicine can improve the degree of depression and sleep quality of patients with depression.
Yang et al., 2019	RCT	CRT	4	763	Music medicine plus Conventional treatment	Conventional treatment	Music medicine had a significant effect on relieving postpartum depression.
Q. Tang et al., 2020	RCT	CRT	55	3984	Music medicine plus Conventional treatment	Conventional treatment	A different effect of music medicine on depression was observed, and the effect might be affected by the therapy process.
Y. X. Wang et al., 2020	RCT	CRT	7	737	Music medicine plus Conventional treatment	Conventional treatment	Music medicine can effectively relieve postpartum depression
Yu et al., 2020	RCT	CRT	11	866	Music medicine plus Conventional treatment	Conventional treatment	Five‐element music therapy can improve depression, anxiety symptoms and sleep quality in patients with depression.
Liu et al., 2021	RCT	CRT	8	583	Music medicine plus routine nursing	Routine nursing	Insufficient evidence of five‐element music in intervention of depression
Zhu et al., 2021	RCT	CRT	7	952	Music medicine plus standard treatment	Standard treatment	The effect of five‐element music intervention on prenatal depression and anxiety maybe better than that of ordinary music.
Zhang et al., 2022	RCT	CRT	12	617	Music medicine plus Conventional treatment	Conventional treatment	Music medicine can improve psychological problems of COPD patients
D. Y. Zhong et al., 2022	RCT	CRT	20	1625	Music medicine plus Conventional treatment	Conventional treatment	Music medicine has benefits in improving HDRS/Ham‐D score and symptoms of PSD patients.

Abbreviations: CCT, case‐control study; COPD, Chronic obstructive pulmonary disease; CRT, Cochrane risk‐of‐bias tool; HDRS/Ham‐D, Hamilton Depression Scale; Jadad means Jadad score; N‐RCT, non‐randomized controlled trial; PSD, Post‐stroke depression; RCT, randomized controlled trial.

### 3.2 PRISMA quality evaluation of included SRs

The methodological quality of each of the 18 SRs was evaluated according to 2020 PRISMA guidelines (X. F. Dai et al., 2015; Y. Q. Dai et al., 2016; Dayuan et al., 2022; Fan et al., 2015; Ji et al., 2015; Liu & Ji, 2021; Q. Tang et al., 2020; Tsai et al., 2014; Wan et al., 2018; X. N. Wang & Lin, 2017; Y. X. Wang et al., 2020; Yan et al., 2019; Yang et al., 2019; Yu et al., 2020; Zhang et al., 2022; Zhao et al., 2016; Zhu et al., 2021; Zou et al., 2017). The assessment showed that the title, rationale, objectives, eligibility criteria, information sources, selection process, data collection process, study characteristics, risk of bias in studies, results of individual studies, results of syntheses, general interpretation, and limitations of all 18 SRs were fully reported. The abstract, data items, study risk of bias assessment in methods, effect measures in methods, synthesis methods, the methods used to synthesize results and provide a rationale for the choice, the methods used to explore possible causes of heterogeneity among study results, the reporting biases in results, the limitations of the review processes used, and the sources of financial or non‐financial support were mostly reported. Complete search formulas, list and define all other variables for which data were sought, the methods required to prepare the data for presentation or synthesis, the methods used to tabulate or visually display results of individual studies and syntheses, sensitivity analyses, certainty assessment, cite studies which were excluded, present results of all investigations of possible causes of heterogeneity among study results, present results of all sensitivity analyses conducted to assess the robustness of the synthesized results, certainty of evidence, the information of protocol, competing interests, and availability of materials were rarely reported. Detailed evaluation results are shown in Table 2.

**TABLE 2:** Preferred Reporting Items for Systematic Reviews and Meta‐Analyses (PRISMA) quality evaluation of included studies.

Section	Topic	Checklist item	Meet all conditions		Partial conditions met		No conditions met	
			Number of included studies	Proportion(%)	Number of included studies	Proportion(%)	Number of included studies	Proportion(%)
Title	Title	Identify the report as a systematic review.	18 (Tsai et al., 2014; Fan et al., 2015; X. F. Dai et al., 2015; Ji et al., 2015; Y. Q. Dai et al., 2016; Zhao et al., 2016; X. N. Wang & Lin, 2017; Zou et al., 2017; Wan et al., 2018; Yan et al., 2019; Yang et al., 2019; Q. Tang et al., 2020; Y. X. Wang et al., 2020; Yu et al., 2020; Liu & Ji, 2021; Zhu et al., 2021; Zhang et al., 2022; Dayuan et al., 2022)	100	0	0	0	0
Abstract	Abstract	structured abstract.	17 (Tsai et al., 2014; Fan et al., 2015; X. F. Dai et al., 2015; Ji et al., 2015; Y. Q. Dai et al., 2016; Zhao et al., 2016; X. N. Wang & Lin, 2017; Zou et al., 2017; Wan et al., 2018; Yan et al., 2019; Q. Tang et al., 2020; Y. X. Wang et al., 2020; Yu et al., 2020; Liu & Ji, 2021; Zhu et al., 2021; Zhang et al., 2022; Dayuan et al., 2022)	94.44	0	0	1 (Yang et al., 2019)	5.56
Introduction	Rationale	Describe the rationale for the review in the context of existing knowledge.	18 (Tsai et al., 2014; Fan et al., 2015; X. F. Dai et al., 2015; Ji et al., 2015; Y. Q. Dai et al., 2016; Zhao et al., 2016; X. N. Wang & Lin, 2017; Zou et al., 2017; Wan et al., 2018; Yan et al., 2019; Yang et al., 2019; Q. Tang et al., 2020; Y. X. Wang et al., 2020; Yu et al., 2020; Liu & Ji, 2021; Zhu et al., 2021; Zhang et al., 2022; Dayuan et al., 2022)	100	0	0	0	0
	Objectives	Provide an explicit statement of the objective(s) or question(s) the review addresses.	18 (Tsai et al., 2014; Fan et al., 2015; X. F. Dai et al., 2015; Ji et al., 2015; Y. Q. Dai et al., 2016; Zhao et al., 2016; X. N. Wang & Lin, 2017; Zou et al., 2017; Wan et al., 2018; Yan et al., 2019; Yang et al., 2019; Q. Tang et al., 2020; Y. X. Wang et al., 2020; Yu et al., 2020; Liu & Ji, 2021; Zhu et al., 2021; Zhang et al., 2022; Dayuan et al., 2022)	100	0	0	0	0
Methods	Eligibility criteria	Specify the inclusion and exclusion criteria for the review and how studies were grouped for the syntheses.	18 (Tsai et al., 2014; Fan et al., 2015; X. F. Dai et al., 2015; Ji et al., 2015; Y. Q. Dai et al., 2016; Zhao et al., 2016; X. N. Wang & Lin, 2017; Zou et al., 2017; Wan et al., 2018; Yan et al., 2019; Yang et al., 2019; Q. Tang et al., 2020; Y. X. Wang et al., 2020; Yu et al., 2020; Liu & Ji, 2021; Zhu et al., 2021; Zhang et al., 2022; Dayuan et al., 2022)	100	0	0	0	0
	Information sources	Specify all databases, registers, websites, organizations, reference lists and other sources searched or consulted to identify studies. Specify the date when each source was last searched or consulted.	18 (Tsai et al., 2014; Fan et al., 2015; X. F. Dai et al., 2015; Ji et al., 2015; Y. Q. Dai et al., 2016; Zhao et al., 2016; X. N. Wang & Lin, 2017; Zou et al., 2017; Wan et al., 2018; Yan et al., 2019; Yang et al., 2019; Q. Tang et al., 2020; Y. X. Wang et al., 2020; Yu et al., 2020; Liu & Ji, 2021; Zhu et al., 2021; Zhang et al., 2022; Dayuan et al., 2022)	100.00	0	0.00	0	0
	Search strategy	Present the full search strategies for all databases, registers and websites, including any filters and limits used.	2 (Wan et al., 2018; Yang et al., 2019)	11.11	6 (Tsai et al., 2014; Y. Q. Dai et al., 2016; Liu & Ji, 2021; Zhu et al., 2021; Zhang et al., 2022; Dayuan et al., 2022)	33.33	10 (Fan et al., 2015;X. F. Dai et al., 2015; Ji et al., 2015; Zhao et al., 2016; X. N. Wang & Lin, 2017; Zou et al., 2017; Yan et al., 2019; Q. Tang et al., 2020; Y. X. Wang et al., 2020; Yu et al., 2020)	55.56
	Selection process	Specify the methods used to decide whether a study met the inclusion criteria of the review, including how many reviewers screened each record and each report retrieved, whether they worked independently, and if applicable, details of automation tools used in the process.	18 (Tsai et al., 2014; Fan et al., 2015; X. F. Dai et al., 2015; Ji et al., 2015; Y. Q. Dai et al., 2016; Zhao et al., 2016; X. N. Wang & Lin, 2017; Zou et al., 2017; Wan et al., 2018; Yan et al., 2019; Yang et al., 2019; Q. Tang et al., 2020; Y. X. Wang et al., 2020; Yu et al., 2020; Liu & Ji, 2021; Zhu et al., 2021; Zhang et al., 2022; Dayuan et al., 2022)	100.00	0	0.00	0	0
	Data collection process	Specify the methods used to collect data from reports, including how many reviewers collected data from each report, whether they worked independently, any processes for obtaining or confirming data from study investigators, and if applicable, details of automation tools used in the process.	18 (Tsai et al., 2014; Fan et al., 2015; X. F. Dai et al., 2015; Ji et al., 2015; Y. Q. Dai et al., 2016; Zhao et al., 2016; X. N. Wang & Lin, 2017; Zou et al., 2017; Wan et al., 2018; Yan et al., 2019; Yang et al., 2019; Q. Tang et al., 2020; Y. X. Wang et al., 2020; Yu et al., 2020; Liu & Ji, 2021; Zhu et al., 2021; Zhang et al., 2022; Dayuan et al., 2022)	100.00	0	0.00	0	0
	Data items	List and define all outcomes for which data were sought. Specify whether all results that were compatible with each outcome domain in each study were sought (e.g., for all measures, time points, analyses), and if not, the methods used to decide which results to collect.	16 (Fan et al., 2015; X. F. Dai et al., 2015; Y. Q. Dai et al., 2016; Zhao et al., 2016; X. N. Wang & Lin, 2017; Zou et al., 2017; Wan et al., 2018; Yan et al., 2019; Yang et al., 2019; Q. Tang et al., 2020; Y. X. Wang et al., 2020; Yu et al., 2020; Liu & Ji, 2021; Zhu et al., 2021; Zhang et al., 2022; Dayuan et al., 2022)	88.89	0	0	2 (Tsai et al., 2014; Ji et al., 2015)	11.11
		List and define all other variables for which data were sought (e.g., participant and intervention characteristics, funding sources). Describe any assumptions made about any missing or unclear information.	5 (X. F. Dai et al., 2015; Y. Q. Dai et al., 2016; Zhao et al., 2016; Y. X. Wang et al., 2020; Liu & Ji, 2021)	27.78	9 (Tsai et al., 2014; Fan et al., 2015; Wan et al., 2018; Yan et al., 2019; Yang et al., 2019; Q. Tang et al., 2020; Yu et al., 2020; Zhu et al., 2021; Dayuan et al., 2022)	50.00	4 (Ji et al., 2015; X. N. Wang & Lin, 2017; Zou et al., 2017; Zhang et al., 2022)	22.22
	Study risk of bias assessment	Specify the methods used to assess risk of bias in the included studies, including details of the tool(s) used, how many reviewers assessed each study and whether they worked independently, and if applicable, details of automation tools used in the process.	17 (Tsai et al., 2014; Fan et al., 2015; X. F. Dai et al., 2015; Ji et al., 2015; Y. Q. Dai et al., 2016; Zhao et al., 2016; Zou et al., 2017; Wan et al., 2018; Yan et al., 2019; Yang et al., 2019; Q. Tang et al., 2020; Y. X. Wang et al., 2020; Yu et al., 2020; Liu & Ji, 2021; Zhu et al., 2021; Zhang et al., 2022; Dayuan et al., 2022)	94.44	0	0	1 (X. N. Wang & Lin, 2017)	5.56
	Effect measures	Specify for each outcome the effect measure(s) (e.g., risk ratio, mean difference) used in the synthesis or presentation of results.	16 (Tsai et al., 2014; Fan et al., 2015; X. F. Dai et al., 2015; Ji et al., 2015; Zhao et al., 2016; Zou et al., 2017; Wan et al., 2018; Yan et al., 2019; Yang et al., 2019; Q. Tang et al., 2020; Y. X. Wang et al., 2020; Yu et al., 2020; Liu & Ji, 2021; Zhu et al., 2021; Zhang et al., 2022; Dayuan et al., 2022)	88.89	0	0	2 (Y. Q. Dai et al., 2016; X. N. Wang & Lin, 2017)	11.11
	Synthesis methods	Describe the processes used to decide which studies were eligible for each synthesis (e.g., tabulating the study intervention characteristics and comparing against the planned groups for each synthesis (item #5)).	16 (Tsai et al., 2014; Fan et al., 2015; X. F. Dai et al., 2015; Ji et al., 2015; Zhao et al., 2016; Zou et al., 2017; Wan et al., 2018; Yan et al., 2019; Yang et al., 2019; Q. Tang et al., 2020; Y. X. Wang et al., 2020; Yu et al., 2020; Liu & Ji, 2021; Zhu et al., 2021; Zhang et al., 2022; Dayuan et al., 2022)	88.89	0	0	2 (Y. Q. Dai et al., 2016; X. N. Wang & Lin, 2017)	11.11
		Describe any methods required to prepare the data for presentation or synthesis, such as handling of missing summary statistics, or data conversions.	2 (Tsai et al., 2014; Dayuan et al., 2022)	11.11	0	0	16 (Fan et al., 2015; X. F. Dai et al., 2015; Ji et al., 2015; Y. Q. Dai et al., 2016; Zhao et al., 2016; X. N. Wang & Lin, 2017; Zou et al., 2017; Wan et al., 2018; Yan et al., 2019; Yang et al., 2019; Q. Tang et al., 2020; Y. X. Wang et al., 2020; Yu et al., 2020; Liu & Ji, 2021; Zhu et al., 2021; Zhang et al., 2022)	88.89
		Describe any methods used to tabulate or visually display results of individual studies and syntheses.	1 (Yu et al., 2020)	5.56	4 (Wan et al., 2018; Yang et al., 2019; Q. Tang et al., 2020; Zhu et al., 2021)	22.22	13 (Tsai et al., 2014; Fan et al., 2015; X. F. Dai et al., 2015; Ji et al., 2015; Y. Q. Dai et al., 2016; Zhao et al., 2016; X. N. Wang & Lin, 2017; Zou et al., 2017; Yan et al., 2019; Y. X. Wang et al., 2020; Liu & Ji, 2021; Zhang et al., 2022; Dayuan et al., 2022)	68.42
		Describe any methods used to synthesize results and provide a rationale for the choice(s). If meta‐analysis was performed, describe the model(s), method(s) to identify the presence and extent of statistical heterogeneity, and software package(s) used.	16 (Tsai et al., 2014; Fan et al., 2015; X. F. Dai et al., 2015; Ji et al., 2015; Zhao et al., 2016; Zou et al., 2017; Wan et al., 2018; Yan et al., 2019; Yang et al., 2019; Q. Tang et al., 2020; Y. X. Wang et al., 2020; Yu et al., 2020; Liu & Ji, 2021; Zhu et al., 2021; Zhang et al., 2022; Dayuan et al., 2022)	88.89	0	0	2 (Y. Q. Dai et al., 2016; X. N. Wang & Lin, 2017)	11.11
		Describe any methods used to explore possible causes of heterogeneity among study results (e.g., subgroup analysis, meta‐regression).	10 (Tsai et al., 2014; Fan et al., 2015; X. F. Dai et al., 2015; Ji et al., 2015; Zou et al., 2017; Wan et al., 2018; Q. Tang et al., 2020; Y. X. Wang et al., 2020; Zhu et al., 2021; Dayuan et al., 2022)	55.56	0	0	8 (Y. Q. Dai et al., 2016; Zhao et al., 2016; X. N. Wang & Lin, 2017; Yan et al., 2019; Yang et al., 2019; Yu et al., 2020; Liu & Ji, 2021; Zhang et al., 2022)	44.45
		Describe any sensitivity analyses conducted to assess robustness of the synthesized results.	8 (Tsai et al., 2014; Fan et al., 2015; X. F. Dai et al., 2015; Zou et al., 2017; Wan et al., 2018; Yang et al., 2019; Q. Tang et al., 2020; Dayuan et al., 2022)	44.45	0	0	10 (Ji et al., 2015; Y. Q. Dai et al., 2016; Zhao et al., 2016; X. N. Wang & Lin, 2017; Yan et al., 2019; Y. X. Wang et al., 2020; Yu et al., 2020; Liu & Ji, 2021; Zhu et al., 2021; Zhang et al., 2022)	55.56
	Reporting bias assessment	Describe any methods used to assess risk of bias due to missing results in a synthesis (arising from reporting biases).	7 (Tsai et al., 2014; Zhao et al., 2016; Wan et al., 2018; Yang et al., 2019; Q. Tang et al., 2020; Zhu et al., 2021; Dayuan et al., 2022)	38.89	0	0	11 (Fan et al., 2015; X. F. Dai et al., 2015; Ji et al., 2015; Y. Q. Dai et al., 2016; X. N. Wang & Lin, 2017; Zou et al., 2017; Yan et al., 2019; Y. X. Wang et al., 2020; Yu et al., 2020; Liu & Ji, 2021; Zhang et al., 2022)	61.11
	Certainty assessment	Describe any methods used to assess certainty (or confidence) in the body of evidence for an outcome.	1 (Zhu et al., 2021)	5.56	0	0	17 (Tsai et al., 2014; Fan et al., 2015; X. F. Dai et al., 2015; Ji et al., 2015; Y. Q. Dai et al., 2016; Zhao et al., 2016; X. N. Wang & Lin, 2017; Zou et al., 2017; Wan et al., 2018; Yan et al., 2019; Yang et al., 2019; Q. Tang et al., 2020; Y. X. Wang et al., 2020; Yu et al., 2020; Liu & Ji, 2021; Zhang et al., 2022; Dayuan et al., 2022)	94.44
Results	Study selection	Describe the results of the search and selection process, from the number of records identified in the search to the number of studies included in the review, ideally using a flow diagram.	9 (Ji et al., 2015; Y. Q. Dai et al., 2016; Zhao et al., 2016; Yang et al., 2019; Q. Tang et al., 2020; Y. X. Wang et al., 2020; Yu et al., 2020; Zhu et al., 2021; Dayuan et al., 2022)	50.00	8 (Fan et al., 2015; X. F. Dai et al., 2015; X. N. Wang & Lin, 2017; Zou et al., 2017; Wan et al., 2018; Yan et al., 2019; Liu & Ji, 2021; Zhang et al., 2022)	44.45	1 (Tsai et al., 2014)	5.56
		Cite studies that might appear to meet the inclusion criteria, but which were excluded, and explain why they were excluded.	0	0	12 (Fan et al., 2015; Ji et al., 2015; Y. Q. Dai et al., 2016; Zhao et al., 2016; Yan et al., 2019; Yang et al., 2019; Q. Tang et al., 2020; Y. X. Wang et al., 2020; Yu et al., 2020; Zhu et al., 2021; Zhang et al., 2022; Dayuan et al., 2022)	66.67	6 (Tsai et al., 2014; X. F. Dai et al., 2015; X. N. Wang & Lin, 2017; Zou et al., 2017; Wan et al., 2018; Liu & Ji, 2021)	33.33
	Study characteristics	Cite each included study and present its characteristics.	18 (Tsai et al., 2014; Fan et al., 2015; X. F. Dai et al., 2015; Ji et al., 2015; Y. Q. Dai et al., 2016; Zhao et al., 2016; X. N. Wang & Lin, 2017; Zou et al., 2017; Wan et al., 2018; Yan et al., 2019; Yang et al., 2019; Q. Tang et al., 2020; Y. X. Wang et al., 2020; Yu et al., 2020; Liu & Ji, 2021; Zhu et al., 2021; Zhang et al., 2022; Dayuan et al., 2022)	100	0	0	0	0
	Risk of bias in studies	Present assessments of risk of bias for each included study.	18 (Tsai et al., 2014; Fan et al., 2015; X. F. Dai et al., 2015; Ji et al., 2015; Y. Q. Dai et al., 2016; Zhao et al., 2016; X. N. Wang & Lin, 2017; Zou et al., 2017; Wan et al., 2018; Yan et al., 2019; Yang et al., 2019; Q. Tang et al., 2020; Y. X. Wang et al., 2020; Yu et al., 2020; Liu & Ji, 2021; Zhu et al., 2021; Zhang et al., 2022; Dayuan et al., 2022)	100	0	0	0	0
	Results of individual studies	For all outcomes, present, for each study: (a) summary statistics for each group (where appropriate) and (b) an effect estimate and its precision (e.g., confidence/credible interval), ideally using structured tables or plots.	18 (Tsai et al., 2014; Fan et al., 2015; X. F. Dai et al., 2015; Ji et al., 2015; Y. Q. Dai et al., 2016; Zhao et al., 2016; X. N. Wang & Lin, 2017; Zou et al., 2017; Wan et al., 2018; Yan et al., 2019; Yang et al., 2019; Q. Tang et al., 2020; Y. X. Wang et al., 2020; Yu et al., 2020; Liu & Ji, 2021; Zhu et al., 2021; Zhang et al., 2022; Dayuan et al., 2022)	100	0	0	0	0
	Results of syntheses	For each synthesis, briefly summarize the characteristics and risk of bias among contributing studies.	18 (Tsai et al., 2014; Fan et al., 2015; X. F. Dai et al., 2015; Ji et al., 2015; Y. Q. Dai et al., 2016; Zhao et al., 2016; X. N. Wang & Lin, 2017; Zou et al., 2017; Wan et al., 2018; Yan et al., 2019; Yang et al., 2019; Q. Tang et al., 2020; Y. X. Wang et al., 2020; Yu et al., 2020; Liu & Ji, 2021; Zhu et al., 2021; Zhang et al., 2022; Dayuan et al., 2022)	100	0	0	0	0
		Present results of all statistical syntheses conducted. If meta‐analysis was done, present for each the summary estimate and its precision (e.g., confidence/credible interval) and measures of statistical heterogeneity. If comparing groups, describe the direction of the effect.	18 (Tsai et al., 2014; Fan et al., 2015; X. F. Dai et al., 2015; Ji et al., 2015; Y. Q. Dai et al., 2016; Zhao et al., 2016; X. N. Wang & Lin, 2017; Zou et al., 2017; Wan et al., 2018; Yan et al., 2019; Yang et al., 2019; Q. Tang et al., 2020; Y. X. Wang et al., 2020; Yu et al., 2020; Liu & Ji, 2021; Zhu et al., 2021; Zhang et al., 2022; Dayuan et al., 2022)	100	0	0	0	0
		Present results of all investigations of possible causes of heterogeneity among study results.	8 (Tsai et al., 2014; Fan et al., 2015; Ji et al., 2015; Zhao et al., 2016; Yan et al., 2019; Q. Tang et al., 2020; Zhu et al., 2021; Dayuan et al., 2022)	44.44	0	0	10 (X. F. Dai et al., 2015; Y. Q. Dai et al., 2016; X. N. Wang & Lin, 2017; Zou et al., 2017; Wan et al., 2018; Yang et al., 2019; Y. X. Wang et al., 2020; Yu et al., 2020; Liu & Ji, 2021; Zhang et al., 2022)	55.56
		Present results of all sensitivity analyses conducted to assess the robustness of the synthesized results.	6 (Tsai et al., 2014; Y. Q. Dai et al., 2016; Yan et al., 2019; Q. Tang et al., 2020; Yu et al., 2020; Zhang et al., 2022)	33.33	0	0	12 (Fan et al., 2015; X. F. Dai et al., 2015; Ji et al., 2015; Zhao et al., 2016; X. N. Wang & Lin, 2017; Zou et al., 2017; Wan et al., 2018; Yang et al., 2019; Y. X. Wang et al., 2020; Liu & Ji, 2021; Zhu et al., 2021; Dayuan et al., 2022)	66.67
	Reporting biases	Present assessments of risk of bias due to missing results (arising from reporting biases) for each synthesis assessed.	10 (Tsai et al., 2014; X. F. Dai et al., 2015; Zhao et al., 2016; Zou et al., 2017; Yan et al., 2019; Q. Tang et al., 2020; Y. X. Wang et al., 2020; Zhu et al., 2021; Zhang et al., 2022; Dayuan et al., 2022)	55.56	0	0	8 (Fan et al., 2015; Ji et al., 2015; Y. Q. Dai et al., 2016; X. N. Wang & Lin, 2017; Wan et al., 2018; Yang et al., 2019; Yu et al., 2020; Liu & Ji, 2021)	44.44
	Certainty of evidence	Present assessments of certainty (or confidence) in the body of evidence for each outcome assessed.	1 (Zhu et al., 2021)	5.56	0	0	17 (Tsai et al., 2014; Fan et al., 2015; X. F. Dai et al., 2015; Ji et al., 2015; Y. Q. Dai et al., 2016; Zhao et al., 2016; X. N. Wang & Lin, 2017; Zou et al., 2017; Wan et al., 2018; Yan et al., 2019; Yang et al., 2019; Q. Tang et al., 2020; Y. X. Wang et al., 2020; Yu et al., 2020; Liu & Ji, 2021; Zhang et al., 2022; Dayuan et al., 2022)	94.44
Discussion	Discussion	Provide a general interpretation of the results in the context of other evidence.	18 (Tsai et al., 2014; Fan et al., 2015; X. F. Dai et al., 2015; Ji et al., 2015; Y. Q. Dai et al., 2016; Zhao et al., 2016; X. N. Wang & Lin, 2017; Zou et al., 2017; Wan et al., 2018; Yan et al., 2019; Yang et al., 2019; Q. Tang et al., 2020; Y. X. Wang et al., 2020; Yu et al., 2020; Liu & Ji, 2021; Zhu et al., 2021; Zhang et al., 2022; Dayuan et al., 2022)	100	0	0	0	0
		Discuss any limitations of the evidence included in the review.	18 (Tsai et al., 2014; Fan et al., 2015; X. F. Dai et al., 2015; Ji et al., 2015; Y. Q. Dai et al., 2016; Zhao et al., 2016; X. N. Wang & Lin, 2017; Zou et al., 2017; Wan et al., 2018; Yan et al., 2019; Yang et al., 2019; Q. Tang et al., 2020; Y. X. Wang et al., 2020; Yu et al., 2020; Liu & Ji, 2021; Zhu et al., 2021; Zhang et al., 2022; Dayuan et al., 2022)	100	0	0	0	0
		Discuss any limitations of the review processes used.	16 (Fan et al., 2015; X. F. Dai et al., 2015; Ji et al., 2015; Y. Q. Dai et al., 2016; X. N. Wang & Lin, 2017; Zou et al., 2017; Wan et al., 2018; Yan et al., 2019; Yang et al., 2019; Q. Tang et al., 2020; Y. X. Wang et al., 2020; Yu et al., 2020; Liu & Ji, 2021; Zhu et al., 2021; Zhang et al., 2022; Dayuan et al., 2022)	88.89	0	0	2 (Tsai et al., 2014; Zhao et al., 2016)	11.11
		Discuss implications of the results for practice, policy, and future research.	18 (Tsai et al., 2014; Fan et al., 2015; X. F. Dai et al., 2015; Ji et al., 2015; Y. Q. Dai et al., 2016; Zhao et al., 2016; X. N. Wang & Lin, 2017; Zou et al., 2017; Wan et al., 2018; Yan et al., 2019; Yang et al., 2019; Q. Tang et al., 2020; Y. X. Wang et al., 2020; Yu et al., 2020; Liu & Ji, 2021; Zhu et al., 2021; Zhang et al., 2022; Dayuan et al., 2022)	100	0	0	0	0
Other information	Registration and protocol	Provide registration information for the review, including register name and registration number, or state that the review was not registered.	2 (Yang et al., 2019; Dayuan et al., 2022)	11.11	0	0	16 (Tsai et al., 2014; Fan et al., 2015; X. F. Dai et al., 2015; Ji et al., 2015; Y. Q. Dai et al., 2016; Zhao et al., 2016; X. N. Wang & Lin, 2017; Zou et al., 2017; Wan et al., 2018; Yan et al., 2019; Q. Tang et al., 2020; Y. X. Wang et al., 2020; Yu et al., 2020; Liu & Ji, 2021; Zhu et al., 2021; Zhang et al., 2022)	88.89
		Indicate where the review protocol can be accessed, or state that a protocol was not prepared.	2 (Yang et al., 2019; Dayuan et al., 2022)	11.11	0	0	16 (Tsai et al., 2014; Fan et al., 2015; X. F. Dai et al., 2015; Ji et al., 2015; Y. Q. Dai et al., 2016; Zhao et al., 2016; X. N. Wang & Lin, 2017; Zou et al., 2017; Wan et al., 2018; Yan et al., 2019; Q. Tang et al., 2020; Y. X. Wang et al., 2020; Yu et al., 2020; Liu & Ji, 2021; Zhu et al., 2021; Zhang et al., 2022)	88.89
		Describe and explain any amendments to information provided at registration or in the protocol.	2 (Yang et al., 2019; Dayuan et al., 2022)	11.11	0	0	16 (Tsai et al., 2014; Fan et al., 2015; X. F. Dai et al., 2015; Ji et al., 2015; Y. Q. Dai et al., 2016; Zhao et al., 2016; X. N. Wang & Lin, 2017; Zou et al., 2017; Wan et al., 2018; Yan et al., 2019; Q. Tang et al., 2020; Y. X. Wang et al., 2020; Yu et al., 2020; Liu & Ji, 2021; Zhu et al., 2021; Zhang et al., 2022)	88.89
Support	Support	Describe sources of financial or non‐financial support for the review, and the role of the funders or sponsors in the review.	12 (Fan et al., 2015; X. F. Dai et al., 2015; Y. Q. Dai et al., 2016; Zhao et al., 2016; Yan et al., 2019; Yang et al., 2019; Q. Tang et al., 2020; Y. X. Wang et al., 2020; Yu et al., 2020; Liu & Ji, 2021; Zhu et al., 2021; Dayuan et al., 2022)	66.67	0	0	6 (Tsai et al., 2014; Ji et al., 2015; X. N. Wang & Lin, 2017; Zou et al., 2017; Wan et al., 2018; Zhang et al., 2022)	33.33
Competing interests	Competing interests	Declare any competing interests of review authors.	6 (Tsai et al., 2014; Zhao et al., 2016; Yang et al., 2019; Q. Tang et al., 2020; Zhu et al., 2021; Dayuan et al., 2022)	33.33	0	0	12 (Fan et al., 2015; X. F. Dai et al., 2015; Ji et al., 2015; Y. Q. Dai et al., 2016; X. N. Wang & Lin, 2017; Zou et al., 2017; Wan et al., 2018; Yan et al., 2019; Y. X. Wang et al., 2020; Yu et al., 2020; Liu & Ji, 2021; Zhang et al., 2022)	66.67
Availability of data, code and other materials	Availability of data, code and other materials	Report which of the following are publicly available and where they can be found: template data collection forms; data extracted from included studies; data used for all analyses; analytic code; any other materials used in the review.	3 (Yang et al., 2019; Q. Tang et al., 2020; Dayuan et al., 2022)	16.67	0	0.00	15 (Tsai et al., 2014; Fan et al., 2015; X. F. Dai et al., 2015; Ji et al., 2015; Y. Q. Dai et al., 2016; Zhao et al., 2016; X. N. Wang & Lin, 2017; Zou et al., 2017; Wan et al., 2018; Yan et al., 2019; Y. X. Wang et al., 2020; Yu et al., 2020; Liu & Ji, 2021; Zhu et al., 2021; Zhang et al., 2022)	83.33

### 3.3 Methodological quality evaluation of the included SRs

The methodological quality of 18 SRs was evaluated according to the AMSTAR 2 scale (X. F. Dai et al., 2015; Y. Q. Dai et al., 2016; Dayuan et al., 2022; Fan et al., 2015; Ji et al., 2015; Liu & Ji, 2021; Q. Tang et al., 2020; Tsai et al., 2014; Wan et al., 2018; X. N. Wang & Lin, 2017; Y. X. Wang et al., 2020; Yan et al., 2019; Yang et al., 2019; Yu et al., 2020; Zhang et al., 2022; Zhao et al., 2016; Zhu et al., 2021; Zou et al., 2017). The AMSTAR 2 research team selected seven key items that affect the production of SRs and the validity of their results, namely items 2, 4, 7, 9, 11, 13, and 15 (Yang et al., 2019; Liu et al., 2023). Only two of the 18 SRs included in this paper reported the registration of the research plan (Dayuan et al., 2022; Yang et al., 2019), and the remaining 16 SRs did not report whether the research plan was registered in advance (X. F. Dai et al., 2015; Y. Q. Dai et al., 2016; Fan et al., 2015; Ji et al., 2015; Liu & Ji, 2021; Q. Tang et al., 2020; Tsai et al., 2014; Wan et al., 2018; X. N. Wang & Lin, 2017; Y. X. Wang et al., 2020; Yan et al., 2019; Yu et al., 2020; Zhang et al., 2022; Zhao et al., 2016; Zhu et al., 2021; Zou et al., 2017). Twelve SRs reported in detail the reasons for exclusion of literature (Y. Q. Dai et al., 2016; Dayuan et al., 2022; Fan et al., 2015; Ji et al., 2015; Q. Tang et al., 2020; Y. X. Wang et al., 2020; Yan et al., 2019; Yang et al., 2019; Yu et al., 2020; Zhang et al., 2022; Zhao et al., 2016; Zhu et al., 2021), and the other nine SRs did not (X. F. Dai et al., 2015; Liu & Ji, 2021; Tsai et al., 2014; Wan et al., 2018; X. N. Wang & Lin, 2017; Zou et al., 2017). Twelve SRs included detailed information on the sources of funding for their SRs (X. F. Dai et al., 2015; Y. Q. Dai et al., 2016; Dayuan et al., 2022; Fan et al., 2015; Liu & Ji, 2021; Q. Tang et al., 2020; Y. X. Wang et al., 2020; Yan et al., 2019; Yang et al., 2019; Yu et al., 2020; Zhao et al., 2016; Zhu et al., 2021), and six not (Ji et al., 2015; Tsai et al., 2014; Wan et al., 2018; X. N. Wang & Lin, 2017; Zhang et al., 2022; Zou et al., 2017). Using the AMSTAR 2 criteria, the methodological quality of the SRs can be divided into four grades: high quality (≤1nonkey item not satisfied); medium quality (>1 noncritical item not satisfied); low quality (one key item not satisfied, with or without nonkey item not satisfied); and very low quality (>1 key item is not satisfied, and with or without nonkey items are not satisfied). The results showed that one SR was rated as high quality (Dayuan et al., 2022), six SRs were rated as low quality (Q. Tang et al., 2020; Tsai et al., 2014; Yan et al., 2019; Yu et al., 2020; Zhang et al., 2022; Zhao et al., 2016), and 11 SRs were rated as very low quality (X. F. Dai et al., 2015; Y. Q. Dai et al., 2016; Fan et al., 2015; Ji et al., 2015; Liu & Ji, 2021; Wan et al., 2018; X. N. Wang & Lin, 2017; Y. X. Wang et al., 2020; Yang et al., 2019; Zhu et al., 2021; Zou et al., 2017). Detailed AMSTAR 2 evaluation results are shown in Table 3.

**TABLE 3:** Methodological quality evaluation results of the included studies.

Study	Item 1	Item 2	Item 3	Item 4	Item 5	Item 6	Item 7	Item 8	Item 9	Item 10	Item 11	Item 12	Item 13	Item 14	Item 15	Item 16	Quality evaluation
Tsai et al., 2014	Y	N	Y	Y	Y	Y	N	Y	Y	N	Y	Y	Y	Y	Y	Y	Low quality
Fan et al., 2015	Y	N	Y	Y	Y	Y	Y	Y	Y	Y	Y	Y	N	Y	N	N	Very low quality
X. F. Dai et al., 2015	Y	N	Y	Y	Y	Y	N	Y	Y	Y	Y	Y	Y	N	Y	N	Very low quality
Ji et al., 2015	Y	N	Y	N	N	N	Y	Y	Y	N	Y	N	N	Y	N	N	Very low quality
Y. Q. Dai et al., 2016	Y	N	Y	Y	Y	Y	Y	Y	Y	Y	N	Y	Y	Y	Y	N	Very low quality
Zhao et al., 2016	Y	N	Y	Y	Y	Y	Y	Y	Y	Y	Y	Y	Y	Y	Y	Y	Low quality
X. N. Wang & Lin, 2017	Y	N	Y	PC	N	N	N	Y	N	N	N	N	N	N	N	N	Very low quality
Zou et al., 2017	Y	N	Y	Y	Y	Y	N	Y	Y	N	Y	N	Y	N	Y	N	Very low quality
Wan et al., 2018	Y	N	Y	Y	N	N	N	Y	Y	N	Y	Y	Y	N	N	N	Very low quality
Yan et al. 2019	Y	N	Y	Y	Y	Y	Y	Y	Y	Y	Y	Y	Y	Y	Y	N	Low quality
Yang et al., 2019	Y	Y	Y	Y	Y	Y	Y	Y	Y	Y	Y	Y	N	N	N	Y	Very low quality
Q. Tang et al., 2020	Y	N	Y	Y	Y	Y	Y	Y	Y	Y	Y	Y	Y	Y	Y	Y	Low quality
Y. X. Wang et al., 2020	Y	N	Y	Y	Y	Y	Y	Y	Y	Y	Y	Y	N	N	N	N	Very low quality
Yu et al., 2020	Y	N	Y	Y	Y	Y	Y	Y	Y	Y	Y	Y	Y	Y	Y	N	Low quality
Liu et al., 2021; Liu & Ji, 2021	Y	N	Y	Y	Y	Y	N	Y	Y	Y	Y	N	N	N	N	N	Very low quality
Zhu et al., 2021	Y	N	Y	Y	Y	Y	Y	Y	Y	Y	Y	Y	Y	Y	N	Y	Very low quality
Zhang et al., 2022	Y	N	Y	Y	Y	Y	Y	Y	Y	N	Y	N	Y	N	Y	N	Low quality
D. Y. Zhong et al., 2022; Dayuan et al., 2022	Y	Y	Y	Y	Y	Y	Y	Y	Y	Y	Y	Y	Y	Y	Y	Y	High quality

*Note*: Item 1 represents whether the question and criteria included elements of the population, intervention, comparison, outcome (PICO), and item 2 represents a systematic evaluation of whether or not the evaluation was designed in advance, and whether there are significant differences between the content of the report and the proposed program. Item 3 covers explanation of the choice of study design type, and item 4 indicates whether a comprehensive manuscript retrieval strategy has been used partially in accordance with partial conformity. Item 5 is what is the repeatability of the study screening, Item 6 represents the repeatability of data extraction. Item 7 is for the purpose of listing and proving that the exclusion causes partially comply with partially comply, Item 8 describes in detail the contents of the included study. Item 9 is partially consistent with the use of appropriate methods to assess bias between natal studies. Item 10 provides information on the sources of funding for the included study. Item 11 indicates the suitability of the method for combining results, and item 12 assesses the potential impact of the bias of the natal study on meta‐analysis results and other evidence synthesis: In interpreting and discussing the results of the systematic evaluation, is there an understanding of the bias of the included study. Item 14 represents a reasonable approach that explains or discusses the heterogeneity observed in the evaluation results. Item 15 represents a quantitative merger with full investigation of publication bias and discussion of its possible impact on the evaluation results. Item 16 represents any potential conflict of interest reported, including any funds received for systematic evaluation. Y, yes; N, no; PC, partially consistent.

### 3.4 Evidence evaluation of GRADE criteria

Eight SRs evaluated depressive symptom indicators (Q. Tang et al., 2020; Tsai et al., 2014; X. N. Wang & Lin, 2017; Y. X. Wang et al., 2020; Yang et al., 2019; Zhang et al., 2022; Zhao et al., 2016; Zhu et al., 2021) using nine measures. GRADE evaluation results showed that one study was rated as high quality, six were rated as medium quality, and one was rated as extremely low quality. Five SRs evaluated the treatment efficiency (X. F. Dai et al., 2015; Dayuan et al., 2022; Yan et al., 2019; Yu et al., 2020; Zou et al., 2017). GRADE evaluation results showed that five SRs were rated as medium quality. Seven SRs evaluated the Hamilton depression scale index (X. F. Dai et al., 2015; Dayuan et al., 2022; Fan et al., 2015; Wan et al., 2018; Yan et al., 2019; Yu et al., 2020; Zou et al., 2017). The GRADE evaluation results showed that one SR was rated as high quality, five SRs were rated as medium quality, and one SR was rated as very low quality. Six SRs evaluated the Self‐rating depression scale (SDS) (Y. Q. Dai et al., 2016; Dayuan et al., 2022; Fan et al., 2015; Ji et al., 2015; Liu & Ji, 2021; Yu et al., 2020). GRADE evaluation results showed that all six SRs were rated as medium quality. Detailed GRADE evaluation results are shown in Table 4.

**TABLE 4:** Evidence evaluation results of grading of recommendations, assessment, development, and evaluation (GRADE) criteria of total effective rate.

Study	Index	Measures	Number of included studies	Model	Effect [CI 95%]	Total score
Tsai et al., 2014	Depressive symptoms	Music medicine + standard treatment vs. standard treatment	8	Random	SMD = −0.510 [−0.681, −0.340]	Medium quality①⑤
Zhao et al., 2016	Depressive symptoms	Music medicine + standard treatment vs. standard treatment	10	Random	SMD = −1.02 [−0.87, −1.17]	Medium quality①⑤
X. N. Wang & Lin, 2017	Depressive symptoms	Music medicine plus routine nursing vs. routine nursing	4	Random	WMD = −2.25 [−3.36, −1.15]	Medium quality①⑤
Yang et al., 2019	Depressive symptoms	Music medicine + conventional treatment vs. conventional treatment	4	Random	SMD = −0.87 [−1.23, −0.51]	Medium quality②⑤
Q. Tang et al., 2020	Depressive symptoms	Music medicine + conventional treatment vs. conventional treatment	17	Random	SMD = −0.66 [−0.86, −0.46]	High quality
Y. X. Wang et al., 2020	Depressive symptoms	Music medicine + conventional treatment vs. conventional treatment	6	Random	SMD = −1.17 [−1.73, 0.61]	Very low quality①②④⑤
Zhu et al., 2021	Depressive symptoms	Music medicine + standard treatment vs. standard treatment	7	Random	SMD = −1.35 [−1.87, −0.84]	Medium quality①⑤
Zhang et al., 2022	Depressive symptoms	Music medicine + conventional treatment vs. conventional treatment	5	Random	SMD= − 1.76 [−3.22, −030]	Medium quality①⑤
X. F. Dai et al., 2015	Effective rate	Music medicine + conventional treatment vs. conventional treatment	5	Fixed	OR = 4.08 [2.28, 7.30]	Medium quality①⑤
Zou et al., 2017	Effective rate	Music medicine + conventional treatment vs. conventional treatment	6	Fixed	OR = 5.69 [3.33, 9.73]	Medium quality①⑤
Yan et al., 2019	Effective rate	Music medicine plus routine nursing vs. routine nursing	4	Fixed	RR = 1.45 [1.23, 1.71]	Medium quality①⑤
Yu et al., 2020	Effective rate	Music medicine + conventional treatment vs. conventional treatment	6	Fixed	OR = 2.80 [1.84, 4.25]	Medium quality①⑤
D. Y. Zhong et al., 2022	Effective rate	Music medicine + conventional treatment vs. conventional treatment	6	Random	OR = 4.02 [2.41, 6.72]	Medium quality①⑤
Fan et al., 2015	Hamilton depression scale	Music medicine + conventional treatment vs. conventional treatment	4	Random	SMD = −1.128 [−2.504, 0.247]	Very low quality①②④⑤
X. F. Dai et al., 2015	Hamilton depression scale	Music medicine + conventional treatment vs. conventional treatment	5	Fixed	OR = 4.08 [2.28, 7.30]	Medium quality①⑤
Zou et al., 2017	Hamilton depression scale	Music medicine + conventional treatment vs. conventional treatment	5	Random	SMD = −3.30 [−5.57, −1.03]	Medium quality①⑤
Wan et al., 2018	Hamilton depression scale	Music medicine + conventional treatment vs. conventional treatment	16	Random	SMD = −4.14 [−6.62, −1.66]	Medium quality①⑤
Yan et al., 2019	Hamilton depression scale	Music medicine plus routine nursing vs. routine nursing	7	Random	SMD = −0.47 [−0.90, −0.04]	Medium quality①⑤
Yu et al., 2020	Hamilton depression scale	Music medicine + conventional treatment vs. conventional treatment	2	Fixed	SMD = 7.65 [−11.11, −4.18]	Medium quality①⑤
D. Y. Zhong et al., 2022	Hamilton depression scale	Music medicine + conventional treatment vs. conventional treatment	14	Random	MD = −3.32 [− 4.27, −2.37]	High quality ①
Fan et al., 2015	Self‐rating depression scale	Music medicine + conventional treatment vs. conventional treatment	4	Random	SMD = −1.223 [−1.655, −0.791]	Medium quality①⑤
Ji et al., 2015	Self‐rating depression scale	Music medicine vs. quiet environment	4	Fixed	SMD = −0.86 [−1.13, −0.58]	Medium quality①⑤
Y. Q. Dai et al., 2016	Self‐rating depression scale	Music medicine + conventional treatment vs. conventional treatment	2	Random	WMD = −10.81 [−12.14, −8.41]	Medium quality①⑤
Yu et al., 2020	Self‐rating depression scale	Music medicine + conventional treatment vs. conventional treatment	2	Random	SMD = −6.14 [−7.97, −4.31]	Medium quality①⑤
Liu et al., 2021	Self‐rating depression scale	Music medicine + conventional treatment vs. conventional treatment	8	Random	SMD = −7.12 [−8.95, −5.28]	Medium quality①⑤
D. Y. Zhong et al., 2022	Self‐rating depression scale	Music medicine + conventional treatment vs. conventional treatment	7	Random	MD = −5.67 [−7.01, −4.32]	Medium quality①⑤

*Note*: ①: the limitations of the research, ②: the inconsistency of the research results, ③: indirect evidence, ④: precision, ⑤: publication bias.

Abbreviations: MD, mean difference; OR, odds ratios; RR, relative risks; SMD, Standard Mean Difference; WMD, Weighted Mean Difference.

## 4 DISCUSSION

### 4.1 The primary findings of this study

SRs are widely recognized as the best evidence synthesis studies in clinical decision‐making (Cook et al., 1997). Therefore, it is highly necessary to conduct SERE in order to better utilize SR as a tool and provide superior evidence for clinical practice. In this study, a total of 18 SRs on music as an intervention for depression were included. The quality and evidence level of these reviews were assessed using 2020 PRISMA guidelines, AMSTAR 2, and GRADE.

#### 4.1.1 The methodological quality of SRs on music interventions for depression needs improvement

This study evaluated the methodological quality of the included literature using PRISMA and AMSTAR 2. The results showed that among the 18 included articles, both PRISMA and AMSTAR 2 identified numerous missing items, indicating poor reporting quality of these SRs. In the methods section, PRISMA scoring primarily focused on the literature search process, literature screening process, and description of statistical methods. We found that only two studies detailed the search strategies for each database (Wan et al., 2018; Yang et al., 2019), while most studies merely described the search terms, with some listing search strategies for individual databases. Although all 18 articles detailed the processes of literature screening and data extraction, only three articles detailed the names of the researchers involved in these operations (Y. Q. Dai et al., 2016; Q. Tang et al., 2020; Zhu et al., 2021). Few SRs provided detailed explanations regarding handling of missing data, subgroup analyses, sensitivity analyses, and grading of evidence. In the appendices, only two SRs detailed protocol mentions (Dayuan et al., 2022; Yang et al., 2019). Additionally, Chinese SRs rarely described conflict of interest and data accessibility statements. Results from AMSTAR 2 similarly showed a bias in the reporting quality of the 18 SRs. One SR was rated as high quality (Dayuan et al., 2022), six as low quality (Q. Tang et al., 2020; Tsai et al., 2014; Yan et al., 2019; Yu et al., 2020; Zhang et al., 2022; Zhao et al., 2016), and 11 as very low quality (X. F. Dai et al., 2015; Y. Q. Dai et al., 2016; Fan et al., 2015; Ji et al., 2015; Liu & Ji, 2021; Wan et al., 2018; X. N. Wang & Lin, 2017; Y. X. Wang et al., 2020; Yang et al., 2019; Zhu et al., 2021; Zou et al., 2017). The deductions primarily concentrated on items 2, 14, 15, and 16. Among the 18 articles included in this study, only two reported registration of study protocols (Dayuan et al., 2022; Yang et al., 2019), while the remaining 16 did not mention whether study protocols were registered in advance (X. F. Dai et al., 2015; Y. Q. Dai et al., 2016; Fan et al., 2015; Ji et al., 2015; Liu & Ji, 2021; Q. Tang et al., 2020; Tsai et al., 2014; Wan et al., 2018; X. N. Wang & Lin, 2017; Y. X. Wang et al., 2020; Yan et al., 2019; Yu et al., 2020; Zhang et al., 2022; Zhao et al., 2016; Zhu et al., 2021; Zou et al., 2017). Few studies extensively analyzed heterogeneity and publication bias on the conclusions of SRs. Most studies did not detail the funding sources and potential conflicts of interest for their included articles. These results are consistent with those of PRISMA, indicating flaws in the design process of these SRs.

#### 4.1.2 The evidence strength of SRs on music interventions for depression is low

The GRADE tool was employed to grade the evidence for each outcome measure of the SRs, evaluating the credibility and reliability of this evidence. The results indicate that for the assessment of four outcome measures, the majority of SRs were rated as moderate and low quality. The primary reasons for downgrading were study limitations and publication bias. Study limitations were the most frequent downgrade factor, manifested in poor methodological quality of most primary studies, such as lack of blinding, inadequate randomization, and insufficient allocation concealment. Despite the increasing number of clinical studies related to music in recent years, most are small‐scale, low‐quality, repetitive studies with a lack of publication of negative results, high heterogeneity among studies, and a shortage of high‐quality evidence from large‐scale multicenter studies.

### 4.2 Evidence of the Efficacy of Music Interventions for Depression

The Beck Depression Inventory is specifically designed to assess the severity of depression (Stepankova Georgi et al., 2019). Developed by the renowned American psychologist Beck AT in the 1960s, it has since been widely utilized in clinical epidemiological surveys. The hamilton depression scale (HAMD) is the most widely used depression assessment scale in clinical practice, employed to evaluate the severity of depression in patients. It is a highly common scale for depression assessment (Berko et al., 2022; Carrozzino et al., 2020; D. Zhong et al., 2023). The SDS consists of 20 items with a 4‐level scoring system, originally developed by W.K. Zung in 1965 (Jokelainen et al., 2019). Its characteristics include ease of use and the ability to intuitively reflect the subjective feelings of depressed patients and changes during treatment. The efficacy of treatment is evaluated from the perspective of the ratio of effectively treated patients. These four indicators have strong guiding significance for the evaluation of depression. This study included 18 SRs, among which eight SRs evaluated depressive symptoms (Q. Tang et al., 2020; Tsai et al., 2014; X. N. Wang & Lin, 2017; Y. X. Wang et al., 2020; Yang et al., 2019; Zhang et al., 2022; Zhao et al., 2016; Zhu et al., 2021). The publication dates of these eight SRs ranged from 2014 to 2022, and each SR included clinical studies that were not entirely overlapping. However, the results of these eight SRs were completely consistent, with all conclusions pointing to the effectiveness of music interventions for depression. Five SRs reported efficacy rates (X. F. Dai et al., 2015; Dayuan et al., 2022; Yan et al., 2019; Yu et al., 2020; Zou et al., 2017). The results showed that the findings of these five SRs were essentially consistent, and the differences were all statistically significant. These results were also evident in the evaluations using HAMD and SDS. Considering the results of these 18 SRs collectively, the effectiveness of music interventions for depression is essentially demonstrated. However, regarding how music can further improve the efficacy of depression treatment, there are currently no more detailed SRs available for reference. Although Zhong investigated differences in improvement of depression based on factors such as duration, frequency, duration of treatment, and volume of music listening, no definitive conclusions were drawn (Dayuan et al., 2022).

## 5 IMPLICATIONS FOR FUTURE RESEARCH

The SRs of music as an intervention for depression published so far have poor methodological quality and low levels of evidence. Additional evaluation is required to determine the efficacy of music as an intervention for depression. More rigorous methodology and comprehensive evaluation of the level of evidence are needed. Future researchers could adopt strict evaluation criteria, such as those described by the Cochrane system, to provide high‐quality, evidence‐based information that clinicians can use to assess the effectiveness of music as an intervention for depression.

## 6 LIMITATIONS

This study has certain limitations. First, the databases available to us are limited. Among the databases searched in this study, PubMed, Web of Science, CNKI, Wanfang, and VIP are the most commonly used databases accessible to us. Other social science databases, such as PsychInfo, Social Service Abstracts, and SocIndex, cannot be accessed due to regional and policy limitations. Although the majority of Chinese social science papers are included in CNKI, Wanfang, and VIP databases, there are still some papers that are not included. Therefore, there may be some papers meeting the inclusion criteria that were overlooked.

Additionally, the music interventions varied across different articles. Some studies investigated receptive music, while others studied recreative music or improvisational music. Even within the category of listening to music, the type of music varied; some studies involved listening to light music, while others focused on classical music. These variations in music approaches contribute to the heterogeneity across studies, and we cannot assess the extent to which these different music modalities may influence the results.

Furthermore, depression was not consistent across studies in this research. Some studies focused on depression in the elderly, while others studied depression in adolescents. Patients also had different comorbidities, with some studies focusing on depression in cancer patients, cardiovascular disease patients, stroke patients, postpartum depression, or patients with chronic obstructive pulmonary disease. The severity of depression also varied, with some studies examining depressive mood while others focused on more severe depression. These different levels of depression further increase the heterogeneity among studies, and we cannot evaluate the extent to which this may affect the results.

## AUTHOR CONTRIBUTIONS

**Dayuan Zhong**: Conceptualization; methodology; software; data curation; writing—review and editing; writing—original draft. **Hui Cheng**: Conceptualization; methodology; software; data curation. **Zhenghua Pan**: Software. **Yumei Liu**: Software. **Pingwen Liu**: Software. **Jiarong Li**: Software. **Jiaqi Chen**: Data curation. **Yihui Deng**: Data curation; conceptualization. **Xueming Ou**: Conceptualization; writing—review and editing. **Huanjie Li**: Writing—review and editing; data curation; conceptualization. **Xiangbo Kong**: Writing—review and editing; conceptualization; data curation.

## CONFLICT OF INTEREST STATEMENT

The authors declare no conflicts of interest.

### PEER REVIEW

The peer review history for this article is available at https://publons.com/publon/10.1002/brb3.3629.

## Data Availability

Requests for additional data may be granted upon reasonable request by contacting the author (dyzhong_medicine@126.com).
